# Supplementary material for: Just another bike? Modelling the interdependence of conventional and electric bicycle ownership and the influence of topography using large-scale travel survey data from Germany
Source: PLoS One. 2025 May 14;20(5):e0322291. doi: 10.1371/journal.pone.0322291 (PMC12077740; doi:10.1371/journal.pone.0322291)
Supplement: Attachment — Submitted filename: 20250131_D_Response_to_reviewers.pdf [file pone.0322291.s002.pdf]

Response to Reviewers

Paper Title: Just another bike? Modelling the interdependence of conventional and electric bicycle ownership and the influence of topography using large-scale travel survey data from Germany

Authors: Leonard Arning, Heather Kaths

Date of Reply: Jan 31st, 2025 (1st revision)

Dear Dr. Michał Suchanek, dear reviewers,

We would like to thank you for taking the time to review our manuscript and for your valuable comments. We have addressed your comments in the new manuscript as laid out below. Our responses are highlighted in dark blue.

We hope that our revisions address your concerns satisfactorily and that the updated manuscript meets your expectations. Please do not hesitate to let us know if further clarifications or adjustments are needed. Thank you again for your valuable feedback and for considering our manuscript.

Kind regards,
Leonard Arning

Reviewers' and Editor's comments with responses:

Journal Requirements:

When submitting your revision, we need you to address these additional requirements.

1. Please ensure that your manuscript meets PLOS ONE's style requirements, including those for file naming. The PLOS ONE style templates can be found at

https://journals.plos.org/plosone/s/file?id=wjVg/PLOSOne_formatting_sample_main_body.pdf and
https://journals.plos.org/plosone/s/file?id=ba62/PLOSOne_formatting_sample_title_authors_affiliations.pdf

> **Response**: We have revised the formatting of our manuscript and the naming of the figure files to adhere to the PLOS ONE style requirements.

2. Thank you for stating the following financial disclosure:

"This research was supported via funding from the German Federal Ministry of Digital and Transport's Bicycle Traffic Endowed Professorship at the University of Wuppertal."

Please state what role the funders took in the study. If the funders had no role, please state: "The funders had no role in study design, data collection and analysis, decision to publish, or preparation of the manuscript." If this statement is not correct you must amend it as needed.

Please include this amended Role of Funder statement in your cover letter; we will change the online submission form on your behalf.

Response: We have clarified our financial disclosure and provide the text for the new statement in the new Cover Letter

3. Please note that in order to use the direct billing option the corresponding author must be affiliated with the chosen institute. Please either amend your manuscript to change the affiliation or corresponding author, or email us at plosone@plos.org with a request to remove this option.

Response: There was a technical issue in the online submission system which did not allow us to select the correct institution for funding. We reached out to PLOS customer care who replied with helpful instructions on how to proceed during the resubmission process, which we will follow.

4. We note that you have indicated that there are restrictions to data sharing for this study. For studies involving human research participant data or other sensitive data, we encourage authors to share de-identified or anonymized data. However, when data cannot be publicly shared for ethical reasons, we allow authors to make their data sets available upon request. For information on unacceptable data access restrictions, please see http://journals.plos.org/plosone/s/data-availability#loc-unacceptable-data-access-restrictions.

Before we proceed with your manuscript, please address the following prompts:

a) If there are ethical or legal restrictions on sharing a de-identified data set, please explain them in detail (e.g., data contain potentially identifying or sensitive patient information, data are owned by a third-party organization, etc.) and who has imposed them (e.g., a Research Ethics Committee or Institutional Review Board, etc.). Please also provide contact information for a data access committee, ethics committee, or other institutional body to which data requests may be sent.

b) If there are no restrictions, please upload the minimal anonymized data set necessary to replicate your study findings to a stable, public repository and provide us with the relevant URLs, DOIs, or accession numbers. Please see http://www.bmj.com/content/340/bmj.c181.long for guidelines on how to de-identify and prepare clinical data for publication. For a list of recommended repositories, please see https://journals.plos.org/plosone/s/recommended-repositories. You also have the option of uploading the data as Supporting Information files, but we would recommend depositing data directly to a data repository if possible.

Please update your Data Availability statement in the submission form accordingly.

Response: We updated our Data Availability statement to now include a more detailed description of the reasons and nature of the access restriction, conditions for data access and contact details.

5. For studies involving third-party data, we encourage authors to share any data specific to their analyses that they can legally distribute. PLOS recognizes, however, that authors may be using third-party data they do not have the rights to share. When third-party data cannot be publicly shared, authors must provide all information necessary for interested researchers to apply to gain access to the data. (https://journals.plos.org/plosone/s/data-availability#loc-acceptable-data-access-restrictions)

For any third-party data that the authors cannot legally distribute, they should include the following information in their Data Availability Statement upon submission:
1) A description of the data set and the third-party source
2) If applicable, verification of permission to use the data set
3) Confirmation of whether the authors received any special privileges in accessing the data that other researchers would not have
4) All necessary contact information others would need to apply to gain access to the data

> **Response**: We updated our Data Availability Statement to now include a more detailed description of the data ownership. We also clarified that we did not receive any special privileges and provided more precise contact information for researchers interested in using the data.

6. We note that Figures 1 and 2 in your submission contain map images which may be copyrighted. All PLOS content is published under the Creative Commons Attribution License (CC BY 4.0), which means that the manuscript, images, and Supporting Information files will be freely available online, and any third party is permitted to access, download, copy, distribute, and use these materials in any way, even commercially, with proper attribution. For these reasons, we cannot publish previously copyrighted maps or satellite images created using proprietary data, such as Google software (Google Maps, Street View, and Earth). For more information, see our copyright guidelines: http://journals.plos.org/plosone/s/licenses-and-copyright.

We require you to either (1) present written permission from the copyright holder to publish these figures specifically under the CC BY 4.0 license, or (2) remove the figures from your submission:

1) You may seek permission from the original copyright holder of Figures 1 and 2 to publish the content specifically under the CC BY 4.0 license.

We recommend that you contact the original copyright holder with the Content Permission Form (http://journals.plos.org/plosone/s/file?id=7c09/content-permission-form.pdf) and the following text:
"I request permission for the open-access journal PLOS ONE to publish XXX under the Creative Commons Attribution License (CCAL) CC BY 4.0 (http://creativecommons.org/licenses/by/4.0/). Please be aware that this license allows unrestricted use and distribution, even commercially, by third parties. Please reply and provide explicit written permission to publish XXX under a CC BY license and complete the attached form."

Please upload the completed Content Permission Form or other proof of granted permissions as an ""Other"" file with your submission.

In the figure caption of the copyrighted figure, please include the following text: "Reprinted from [ref] under a CC BY license, with permission from [name of publisher], original copyright [original copyright year]."

2) If you are unable to obtain permission from the original copyright holder to publish these figures under the CC BY 4.0 license or if the copyright holder's requirements are incompatible with the CC BY 4.0 license, please either i) remove the figure or ii) supply a replacement figure that complies with the CC BY 4.0 license. Please check copyright

information on all replacement figures and update the figure caption with source information. If applicable, please specify in the figure caption text when a figure is similar but not identical to the original image and is therefore for illustrative purposes only.

The following resources for replacing copyrighted map figures may be helpful:

USGS National Map Viewer (public domain): http://viewer.nationalmap.gov/viewer/
The Gateway to Astronaut Photography of Earth (public domain): http://eol.jsc.nasa.gov/sseop/clickmap/
Maps at the CIA (public domain): https://www.cia.gov/library/publications/the-world-factbook/index.html and https://www.cia.gov/library/publications/cia-maps-publications/index.html
NASA Earth Observatory (public domain): http://earthobservatory.nasa.gov/
Landsat: http://landsat.visibleearth.nasa.gov/
USGS EROS (Earth Resources Observatory and Science (EROS) Center) (public domain): http://eros.usgs.gov/#
Natural Earth (public domain): http://www.naturalearthdata.com/

> **Response**: We address this concern in a separate file called "Image copyright declaration" submitted alongside the revised manuscript. The images were created by us without using copyright protected map images or prepublished figures. We only used data that we have permission to use. We clarified the data sources and data licenses used for the creation of these two figures in the figure captions and the references section.

Additional Editor Comments:

Thank you for submitting your manuscript to PLOS ONE. After consideration of the reviews provided by our expert reviewers, I have reached a decision of major revision for your manuscript. Below, I provide a summary of the key points raised by the reviewers to help guide your revisions:

Novelty and Contribution:
While the manuscript is well-written, the reviewers have noted that the degree of novelty and innovation is limited. To strengthen your submission, it is recommended to:
Clearly differentiate your study from existing works using the same dataset (e.g., Kohlrautz and Kuhnimhof).
Highlight the novel contributions of your study explicitly in the introduction, potentially through a dedicated "Main Contribution" section.

Practical Use Cases:
Reviewer 1 suggests that including practical use cases in the discussion section would add value to your study. These could include applications for transportation planners, business models for manufacturers, or other relevant domains such as simulations.

Clarity of Methods:
Reviewer 2 pointed out that the mathematical presentation of the nested logit model and multivariate probit model is not sufficiently clear. Adding more detailed equations and explanations would improve the clarity and rigor of your methodology section.

Figures and Data Visualization:
To enhance the readability and impact of your manuscript, Reviewer 2 recommends adding more comparative figures, particularly to illustrate key differences between conventional and electric bicycles. This would provide a stronger visual representation of your findings.

Formatting and Presentation:

Ensure consistency in formatting, especially in the references section.
Address any minor typographical errors to improve the overall presentation of the manuscript.
Data Accessibility:
Reviewer 1 noted that your manuscript does not fully adhere to the PLOS Data Availability Policy. Please ensure all underlying data are made fully accessible or clearly state any limitations.

I invite you to revise your manuscript in response to these comments. In preparing your revision, please provide a detailed response to each reviewer comment, indicating how you have addressed their concerns. If you choose not to address a specific comment, provide a clear and thorough justification. Please note that while the revisions address several substantial points, they do not necessarily guarantee acceptance, as the revised manuscript will undergo further evaluation.

Thank you for your submission.

> **Response**: Thank you for summarizing the reviewers' comments. We will address them individually below.

[Note: HTML markup is below. Please do not edit.]

Reviewers' comments:

Reviewer's Responses to Questions

Comments to the Author

1. Is the manuscript technically sound, and do the data support the conclusions?

The manuscript must describe a technically sound piece of scientific research with data that supports the conclusions. Experiments must have been conducted rigorously, with appropriate controls, replication, and sample sizes. The conclusions must be drawn appropriately based on the data presented.

Reviewer #1: Yes

Reviewer #2: Yes

---

2. Has the statistical analysis been performed appropriately and rigorously?

Reviewer #1: Yes

Reviewer #2: N/A

---

3. Have the authors made all data underlying the findings in their manuscript fully available?

The PLOS Data policy requires authors to make all data underlying the findings described in their manuscript fully available without restriction, with rare exception (please refer to the Data Availability Statement in the manuscript PDF file). The data should be provided as part of the manuscript or its supporting information, or deposited to a public repository. For example, in addition to summary statistics, the data points behind means, medians and variance measures should be available. If there are restrictions on publicly sharing data—e.g. participant privacy or use of data from a third party—those must be specified.

Reviewer #1: No

Reviewer #2: Yes

Response: See response regarding the updated Data Availability statement.

---

4. Is the manuscript presented in an intelligible fashion and written in standard English?

PLOS ONE does not copyedit accepted manuscripts, so the language in submitted articles must be clear, correct, and unambiguous. Any typographical or grammatical errors should be corrected at revision, so please note any specific errors here.

Reviewer #1: Yes

Reviewer #2: Yes

---

5. Review Comments to the Author

Please use the space provided to explain your answers to the questions above. You may also include additional comments for the author, including concerns about dual publication, research ethics, or publication ethics. (Please upload your review as an attachment if it exceeds 20,000 characters)

Reviewer #1: The paper apply basic models to survey based data (mobility in Germany) and two spatial data (degree of urbanisation and topography) to identify and analyse influential factors for e-bike ownership. The paper is well written, the methodology is clearly explained and the results are well presented.
However, it must be noted that the novelty and degree of innovation is very limited. Standard methods are applied to a data set that has already been analyzed in a similar way in many papers (some of which have been cited). The results clearly show and underline what one would already think influences e-bike ownership. Use cases on how the information can be

used would be interesting and could be a part of the discussion. Apart from business models of bike manufacturers, there are certainly also use cases for transportation planners, simulations, etc. which one could elaborate.

Specific recommendation:
- Clearer differentiation from existing papers that use the same data set to investigate similar questions (e.g. Kohlrautz and Kuhnimhof)

> **Response**: Thank you for pointing out this shortcoming. In the literature review (ll. 129 ff.) we now more explicitly point out how our study expands on the work of Kohlrautz and Kuhnimhof, namely by including gradient and differentiating between c-bikes and e-bikes. Through the changes made in reaction to your next comment, we now explicitly highlight how our work departs from existing literature in general, namely by including gradient, differentiating between c-bikes and e-bikes, and investigating the relationship between the two ownership decisions.

- Clearly emphasize novelty and innovation, if necessary list the "Main Contribution" in the introduction

> **Response**: We now highlight the novel aspects of our research and findings in the Introduction (ll. 54 ff.) and Conclusion (ll. 479 ff.; l. 484).

- Add specific use cases on how and where the results can be applied in discussion

> **Response**: We added substantial discussion on this in the chapter "Suitability of model types and implications for policy and modelling practice", namely ll. 437 ff. and ll. 442 ff.

Reviewer #2: Regarding this manuscript, I have the following suggestions for the author's reference, and I look forward to adding more readability to this study.
1.The formatting is not standardised in some places in the manuscript, such as the references.

> **Response**: Thank you for point out this flaw. We have completely revised the formatting of the manuscript to adhere strictly to the PLOS ONE style guidelines. In particular, we revised the reference section by adding missing information for some sources and follow the PLOS ONE reference style guide more closely, such as using abbreviated journal names, access dates, and more.

2.There are only four figures in the manuscript, which is too few, and some relevant comparative figures of conventional bicycles and e-bikes can be added appropriately to make it easier for readers to understand.

> **Response**: Thank you for giving us the chance to expand on the dynamic development of the electric bicycle market. We have inserted a new, original Figure 1 which visualised the growth of e-bike sales at the cost of conventional bicycles in Germany based on industry data. To allow for a more intuitive understanding of the overall availability of e-bikes in our sample population and the strong influence of the most meaningful sociodemographic variable, namely age, we furthermore provide a new

Figure 5 which visualised the share of c-bike and e-bike ownerships (and combinations of both) across the age cohorts.

3.Too few formulas to introduce the nested logit model and the multivariate probit model, not clear enough.

Response: We substantially expanded the Models section to be more thorough on model derivation and how the models are implemented in our research. For nested logit, we expanded on how a closed form for choice probability is derived, the decomposition of error terms, and how the latter relates to the nesting structure. For multivariate probit, we also expanded on the distribution of the error terms and how it relates to the model parameter R.
